# Quantitative Determination of *Fusarium* *proliferatum* Concentration in Intact Garlic Cloves Using Near-Infrared Spectroscopy

**DOI:** 10.3390/s16071099

**Published:** 2016-07-15

**Authors:** Elena Tamburini, Elisabetta Mamolini, Morena De Bastiani, Maria Gabriella Marchetti

**Affiliations:** Department of Life Science and Biotechnology, University of Ferrara, Via L. Borsari, 46, 44121 Ferrara, Italy; mme@unife.it (E.M.); morena.debastiani@unife.it (M.D.B.); mhm@unife.it (M.G.M.)

**Keywords:** near infrared spectroscopy, *Fusarium proliferatum*, garlic, chemotypization

## Abstract

*Fusarium proliferatum* is considered to be a pathogen of many economically important plants, including garlic. The objective of this research was to apply near-infrared spectroscopy (NIRS) to rapidly determine fungal concentration in intact garlic cloves, avoiding the laborious and time-consuming procedures of traditional assays. Preventive detection of infection before seeding is of great interest for farmers, because it could avoid serious losses of yield during harvesting and storage. Spectra were collected on 95 garlic cloves, divided in five classes of infection (from 1-healthy to 5-very highly infected) in the range of fungal concentration 0.34–7231.15 ppb. Calibration and cross validation models were developed with partial least squares regression (PLSR) on pretreated spectra (standard normal variate, SNV, and derivatives), providing good accuracy in prediction, with a coefficient of determination (R^2^) of 0.829 and 0.774, respectively, a standard error of calibration (SEC) of 615.17 ppb, and a standard error of cross validation (SECV) of 717.41 ppb. The calibration model was then used to predict fungal concentration in unknown samples, peeled and unpeeled. The results showed that NIRS could be used as a reliable tool to directly detect and quantify *F. proliferatum* infection in peeled intact garlic cloves, but the presence of the external peel strongly affected the prediction reliability.

## 1. Introduction

*Fusarium proliferatum* is a world-wide occurring saprophytic fungi, also known to be a causal agent of several diseases for a broad range of economically important plants [1]. Moreover, it is a toxigenic species, producing a group of the most dangerous mycotoxins—fumonisins, causing toxicological effects in animals and plants, as well as in humans [2]. Numerous studies have confirmed the presence of fumonisins in plant material contaminated with their producers, with a dramatic effect on yield and quality of agricultural products [3,4]. *F. proliferatum* is known to be one of the main causes of maize cob fusariosis [5,6], but can also colonize wheat, barley, rice, asparagus, pea, onion, tomato, pineapple, and various palms [7,8,9,10]. Recently, it has also been isolated from uncultivated plants including reed, sorrel, prairie grasses, and pine [11]. Worldwide geographical distribution and a wide range of hosts provide evidence of the extraordinary adaptation ability of the species, enabling it to colonize new environments—often in diverse climatic conditions [12]. Therefore, a sensitive and rapid method for the determination of the grade of fungal contamination in vegetal matrices is required to preserve the quality and safety of food and agricultural commodities in general. The great majority of the already-existing techniques are conceived to either determine mycotoxin content in samples using instrumental chromatography techniques [13], or directly identify the fungal infection by genetic chemotyping via polymerase chain reaction (PCR) assays [14]. Both approaches are accurate and reliable, but difficult and expensive, and not suitable for a real-time response. Additionally, they are time consuming, requiring a well-equipped laboratory and skilled laboratory staff. Moreover, PCR-based techniques have some technical limits due to protocol complexities and the choice of specific primers for each species [15].

In the past few decades, many researchers have focused on the potential use of near-infrared spectroscopy (NIRS), a powerful spectroscopic procedure for the detection of organic compounds in matter [16]. In the field of agriculture, food, medicine, paper, polymers, etc., intense and aggressive interest has been directed toward NIR spectroscopy because it is a fast, nondestructive, environmentally-friendly, and highly accurate method that requires little expert training in routine analysis [17]. 

In recent years, promising results have been obtained applying NIRS methodology to detect mycotoxins and mycotoxigenic fungal contamination in agricultural products, including studies on the estimation of deoxynivalenol, ergosterol, vomitoxin, and fumonisin in single kernels of highly infected wheat, maize, and barley [18,19,20], aflatoxins and ochratoxin A in paprika [21] and red chili powder [22]. Sirisomboon, et al. [23] reported an application of NIR spectroscopy to detect aflatoxigenic fungal infection in rice with a wavelength range between 950 and 1650 nm. Singh, et al. [24] used a short-wave NIR hyperspectral imaging system to detect storage fungi in wheat. 

Overall, the studies described above collectively show the potential for the development of NIR methods for the detection of mycotoxins and mycotoxigenic fungi in agricultural products—particularly cereals and cereal products. However, to the best of the authors’ knowledge, the application of NIR Spectroscopy for the determination of toxigenic fungi in garlic has not been reported so far. 

Garlic is among the oldest known horticultural crops and is grown worldwide. Annual world garlic production is around 23 million tons. China is by far the largest producer of garlic, with around 20 million tons, and Spain has the highest production rate in the European Union and the ninth highest in the world, with approximately 154,000 tons per annum [25]. *F. proliferatum* has been recognized as one of the principal causal agent of clove rot during growth in the field and in storage, leading to serious economic losses [26]. Therefore, a method able to select only the healthy cloves before seeding could be of great interest to farmers. Moreover, the development of a NIRS-based non-destructive and rapid method could represent a step forward in the control of the entire supply chain, up to the market. In fact, once calibrated, NIR instruments could be also properly used in the post-harvest phases to check the healthy status of bulbs during storage and shelf-life.

Thus, the objective of this research was to lay the ground for a NIRS-based quantitative method to measure *F. proliferatum* concentration in intact garlic cloves, and then predict fungal concentration in unknown samples. 

## 2. Materials and Methods

**Figure 1:**
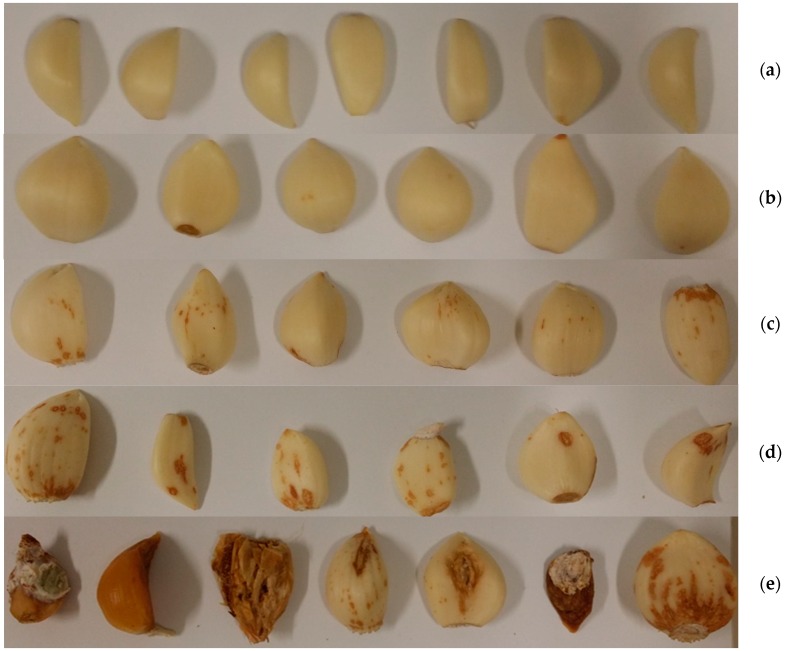
Examples of progressive grades of infection in garlic cloves: (**a**) healthy or asymptomatic; (**b**) slightly infected; (**c**) infected; (**d**) highly infected; (**e**) very highly infected or completely damaged.

*Garlic samples and pretreatments.* White garlic (*Allium sativum* L.) plants were cultivated in 2015 in the Ferrara district of PGI (Protected Geographical Indication) garlic production, located in northeastern Italy. The fresh bulbs, harvested in July, were separated in cloves and deprived of the typical external white/light brown sheath. Bulb harvesting was carried out based on a completely random experimental design, so as to provide a representative sampling of the entire field [27]. Cloves were classified in grade of infection scale from 1 (healthy) to 5 (very highly infected) based on visual observations of symptoms. Symptomatic cloves showed increasing tan-colored rot, evolving in emptied and softened cavities, with necrotic spots in the most severe cases. Examples are reported in Figure 1. A total of 95 peeled cloves were examined, classifying 20 samples for each Class of infection, except for 15 samples in Class 4. Samples were spread on a plastic tray and stored at 4 °C up to submission to NIR spectra collection and analysis in laboratory. 

Then, based on the same sampling procedure, other new 45 samples of garlic cloves were harvested and collected, and divided in two external validation sets: 15 peeled (as those used for calibration) and 30 unpeeled, respectively. 

*NIRS analysis.* FT-NIR diffuse reflectance spectra of garlic cloves samples were collected with a NIRFLex N-500 (Büchi, Flawil, Switzerland), equipped with the Solids Cell Module (Büchi, Flawil, Switzerland) and set up with a polarization interferometer with TeO_2_ wedges. Fresh samples were flattened on the glass surface by using a XL-sample holder 2.5 cm in diameter, suitable for small samples or samples of particular shape (such as garlic cloves). For each clove, three different spectra were acquired, the same sample randomly were re-placed on the sample holder in three different positions. The instrument was designed to be operational for working temperature conditions from 5 up to 35 °C, without any drift of the spectra signal. The reflectance spectra were recorded using NIRW are software version 1.4 (Büchi, Flawil, Switzerland) and scanning the full range—from 1000 to 2500 nm—at 8 nm intervals. Measurements were carried out at 4 scans/s with a wavenumber accuracy of ±0.2 nm (measured with HF gas cell at an ambient temperature of 25 ± 5 °C). To obtain a good signal-to-noise ratio, 16 scans for each spectrum were averaged during each spectral acquisition, resulting in a total measurement time of a few seconds. Every spectrum acquisition was preceded by the acquisition of an internal and external reference to optimize the spectrum baseline.

*Chemometrics*. All chemometric analysis—including calibration and validation—were performed using NIRCal 5.0 (Büchi, Flawil, Switzerland). The wavelengths for each set of processing data were suggested by NIRCal 5.0, based on correlation coefficients calculated for each wavelength [28]. Wavelength selection is carried out by the software based on an iterative procedure (Calibration wizard^®^) combining all possible spectral information and spectra pretreatments to obtain the best combination for calibration.

To establish the relationship between the reference analysis and NIR values, the Partial Least Squares Regression (PLSR) was used. The optimum number of factors to be used was determined by the predicted residual error sum of squares (PRESS) calculation that shows the sum of squares of deviation between predicted and reference values [29]. The selection of the best quantitative regression models was carried out using squared determination coefficient for calibration and cross validation (R^2^), standard error of calibration (SEC), and standard error of cross-validation (SECV). Ratio of Performance to Deviation (RPD)—i.e., the relationship between the SD (Standard Deviation) of the entire population divided by the SEC (or SECV, or SEP), was also calculated for both calibration and cross validation [30]. Cross validation, as default software output, was performed in blockwise mode, splitting the calibration set into three-fold segments, testing one segment as a validation set and the remaining as a calibration set. Quality of calibration was described in the Q-value, calculated by the NIRCal 5.0 software combining all relevant statistical measures (SEC, SEP, regression coefficients). This quality index qualifies the calibrations using a number between 0 (useless) and 1 (ideal). When achieving a Q-value greater than 0.50, the calibration will give reliable results [31]. Outlier detection was performed by the software based on Mahalanobis distance criterion [32]. To test the probability of autocorrelation between a time-series spectra, the Durbin-Watson (DW) test, calculated on residuals of PLSR, was used [33]. More details on NIRS method development can be found in Tamburini, et al. [34]. Finally, the standard error of the laboratory (SEL)—i.e., the error of the reference data—was reported in order to allow a comparison between that value and the NIRS performance (SEC and SECV). 

Raw absorbance spectra were processed with a combination of Standard Normal Variate (SNV) and first derivative (Savitzky–Golay three points), as mathematical pretreatments. SNV is a mathematical transformation method of the spectra used to remove slope variation and to correct for scatter effects [35]. SNV removes the multiplicative interferences of scatter, particle size, and the change of light distance. It corrects both multiplicative and additive scatter effects. First derivative eliminates baseline drifts, and small spectral differences are enhanced. Derivatives in general are mainly used to resolve peak overlap (or enhance resolution) and eliminate constant and linear baseline drift between samples. Spectral first derivative has been calculated here by Savitzky-Golay polynomial fitting, where the data within a moving window are fitted by a polynomial of a given degree to generate a differential of a chosen degree. In this procedure, it is very important to select the proper differentiation width of the moving window in the function. The width should not exceed one point five times the half width of absorbance peak in the spectra [36].

The calibration for *F. proliferatum* concentration was then validated by means of external validation: 45 new samples were acquired to obtain additional data and evaluate the predictive capability of the model. The prediction accuracy was considered in terms of squared correlation coefficient (R^2^) and root mean standard error of prediction between predictions and reference values (RMSEP).

*Identification of* Fusarium proliferatum, *genomic DNA extraction, and fungal biomass quantification in garlic cloves*. The reference *F. proliferatum* strain (s. designation: 1004.1) was grown on Potato-Dextrose-Agar (PDA) medium (FLUKA- Sigma-Aldrich) at room temperature (25 °C) for 5–6 days under black light. The surface is white, becoming purple-violet with age, and the pigment varies in violet intensity. Fungal genomic DNA (gDNA) was extracted and purified from mycelium following the procedure reported in Griffin [37]. To determine *F. proliferatum* concentration in garlic clove samples, each entire clove was weighed and deprived of the outer surface, collecting it in a mortar with liquid nitrogen and ground into fine powder with a sterile pestle. Then, 50–100 micrograms were withdrawn and lysed with I-GENOMIC PLANT Mini Kit (iNtRON Biotechnology, New York, NY, USA). The residue of the lyophilized material not useful for the extraction was stored at −20° C. Quantitative determination of fungal gDNA was obtained by electrophoresis (0.8% *w/v* agarose gel) and spectrophotometry (ND-1000 Nano Drop, Thermo Scientific, Wilmington, NC, USA). Primer design, specificity evaluation, and conventional PCR setting were carried out as reported by [38,39]. Sybr Green real-time PCR was used for evaluating specificity on the gDNA in reference *F. proliferatum* and in diseased garlic tissues. Reactions were performed in a CFX apparatus (CFX96 Touch™ machine—USA-BioRad, Hercules, CA, USA), and the results were analyzed using the manufacturer’s software (CFX Manager Software, v. 3.1; BioRad, Hercules, CA, USA) [40,41,42]. qPCR was performed on two biological replicates of each sample. Standard curves of DNA quantification were obtained from five dilutions of *F. proliferatum* four-fold, starting from 14.3 ng/μL. The amount of fluorescence emission in qPCR correlates to the initial amount of target template during exponential phase [43]. Results are expressed on fresh weight of clove and are related to the total *F. proliferatum* concentration as active biomass and spore present in the sample.

## 3. Results and Discussion

### 3.1. Quantification of F. proliferatum in Garlic Cloves

Absolute quantification yields the exact number of target DNA molecules by comparison with the DNA standards using a calibration curve, and in this case has allowed the quantification of active biomass of *F. proliferatum* in each clove sample. The reliability of the absolute quantification method depends on the PCR efficiency for the target and depends on the calibration curve. An average squared regression (R^2^) of 0.999 and a PCR efficiency of 95% were obtained, and to extract quantification data from any value, the following equation is used: Quantity = 10 ^(Cq−b)/m^, where b is the y-intercept and m is the slope of the linear regression. The quantity (in picograms) was defined by the serial dilutions used to create the standard curve, because all gene data from garlic samples was obtained by interpolating their PCR signals (Cq) into the standard curve.

Chemotypization analysis allowed us to determine the *F. proliferatum* concentration in garlic cloves from its DNA extraction, because from the DNA it is possible to identify specific genes or the microbial species present in a sample. Quantitative results on the 95 samples used as calibration set are reported in Table 1.

As can be noted, at a first glance, different grades of visual infection correspond to a specific fungal concentration interval, even though overlapping of the extreme values occurred. Moreover, the interesting fact is that several samples have been found outside the interval of the class of infection. Namely, the *F. proliferatum* concentration in garlic cloves resulted higher or lower than expected by the visual grade of infection. For higher values, a possible explanation could be found in the fact that visual infection has not yet manifested itself in a manner corresponding to the presence of the fungus. For lower concentrations, the damage manifested is reasonably not exclusively attributable to *F. proliferatum*, but also to other fungal non-toxigenic species commonly present as contaminants in cloves, such as *Aspergillus* spp., *Penicillium* spp., and *Botrytis* spp. [44]. It is worth noting that such effects are more evident at the extreme classes, where the symptoms are absent or highly manifested. Thus, to evaluate the grade of infection of garlic cloves caused by *F. proliferatum*, direct quantification of fungus did not seem to be the most appropriate technique to be associated with the visual grade of infection. 

### 3.2. NIR Spectra Analysis

**Figure 2:**
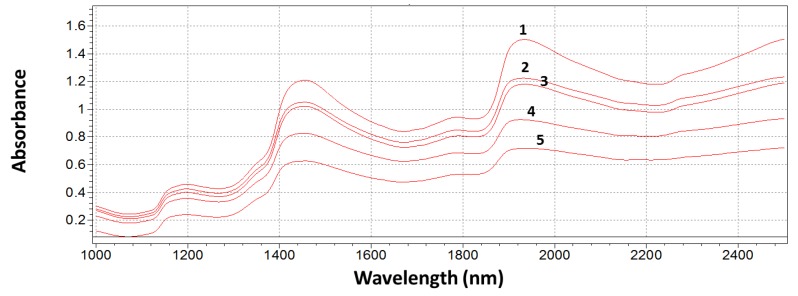
Original raw near-infrared (NIR) spectra of garlic gloves at different grades of infection. Each spectrum represents the average spectrum for its grade of infection.

Examples of original NIR absorption spectra of garlic cloves obtained over a wavelength range between 1000 and 2500 nm are shown in Figure 2. Figure 2 shows the average spectrum for each grade of infection. It is worth noting that absorption decreased in increasing grades of infection, being higher for grade 1 and lower for grade 5, probably due to the progressive lower water content of the sample. 

**Figure 3:**
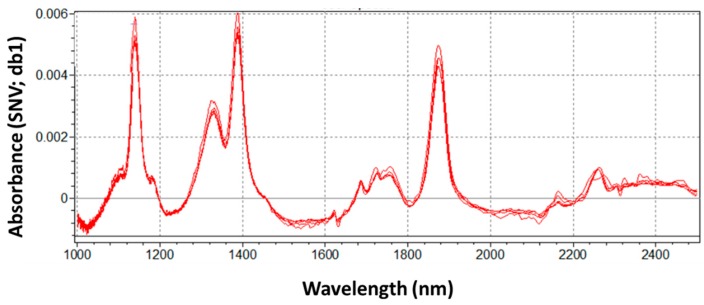
Pretreated NIR average spectra (SNV, Standard Normal Variate; db1, first derivative) of garlic cloves at different grades of infection. Each spectrum represents the average spectrum for its grade of infection.

Typically, spectra of highly complex vegetal materials do not allow easy visual interpretation, because of the presence of several interferences, water content, and light scattering of the sample surface. In these cases, spectra pretreatments are highly recommended to improve peaks resolution (Figure 3) [45].

Significative wavelengths were automatically selected by the software in the range of 1000–2000 nm (as evidenced by the red bar in Figure 1 and Figure 2). Spectra are dominated by peaks at 1150, 1360, 1390–1400, and 1900 nm, respectively, together with a group of weaker signals in the interval 1650–1750 nm. Peaks at 1150 and 1360 nm can be assigned to the second overtone of C–H stretching of CH_3_/CH_2_ groups [46]. These functional groups are present in the chitin molecule—an important cellular compound that contributes to the fungal cell wall. A relevant peak at 1390–1400 nm hides different overlapped signals derived from the second overtone of O–H stretching of water, of aryl–OH of fumonisins, and of pyran-OH of allixin—a phytoallexin present in high concentration in garlic gloves [47]. The first overtone of O-H stretching of water falls at 1900 nm. Different intensity of reflectance (and consequently of absorbance) appears to confirm that moisture in garlic gloves affects the overall extent of fungal infection. The moisture content of substrate is one of the most important factors governing the fungal growth and mycotoxin production [48]. A broadened and highly-overlapped group of weak signals in the interval 1650–1750 nm, combined with a signal at 1900 nm, could correspond to sulfur amino acids (as S-allyl-cisteine) and allyl thiosulfinates (mainly allicin) present in garlic [49]. 

### 3.3. NIRS Model Development

The PLS calibration models were built using the whole spectra (choosing 1251 of a total of 1501 wavelength points theoretically collectible). All spectral regions were used to assess correlations between the absorbance and the associated analytical information, with the exception of the combination bands region beyond 2000 nm automatically excluded from calculation by the software, because they were not correlated with analytical information.

**Figure 4:**
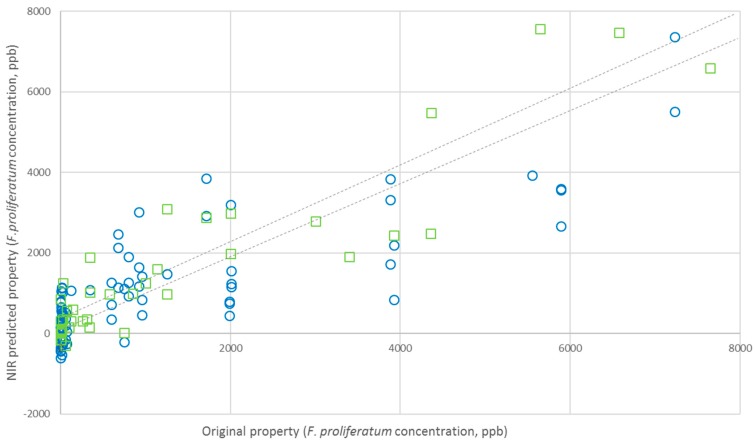
NIRS calibration (blue circle) and validation (green square) curves for *F. proliferatum* concentration (ppb) in peeled garlic cloves.

A total of 285 spectra were collected (three spectra for each sample) and used to develop a calibration model corresponding to the 95 samples of garlic gloves, in the range of *F. proliferatum* concentration 0.34–7231.15 ppb; no outliers were found in the sample set. The blockwise cross-validation was performed by the software by randomly choosing two samples at a time from the calibration set (C-set) to be assigned to the validation set (V-set). Finally, 190 samples were allocated in the C-set and 95 in the V-set. The regression model (four factors) and the cross-validation curve are shown in Figure 4. The standard error of calibration (SEC) and the regression coefficient (R^2^) were 0.012 and 0.983, respectively; the validation samples were predicted with a SECV of 0.016 and a R^2^ of 0.945 (see Table 2). Based on the DW statistics, both the C-set and V-set showed no autocorrelation. RPD for calibration and cross validation were satisfactory in terms of predictive ability of the model. A comparison between SEL and SEC has shown an unavoidable strong worsening of the accuracy when passing from reference assays to NIRS results (real-time PCR technique is known as very precise and accurate), due to the intrinsic characteristic of NIRS to be a secondary technique. In fact, in order to correlate the sample absorbance to the concentration of a specific compound, the accurate amount of the compound under analysis must be known. For this reason, NIRS technologies are initially dependent on other chemical methods (also known as reference methods or primary techniques) to develop a calibration model and validate it properly. Precision and accuracy of NIRS calibrations will be strictly determined by the quality of the reference data, and, principally due to statistical errors propagation, part of them is unavoidably lost. 

The best-performing calibration model—obtained with the PLSR (fitting four factors)—and the cross-validation curve are shown in Figure 4. 

**Figure 5:**
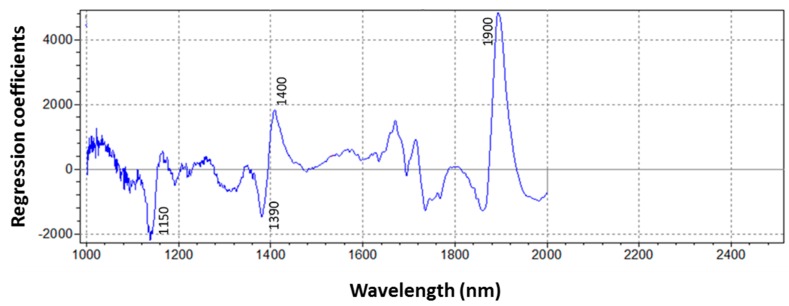
Regression coefficients plot for *F. proliferatum* concentration PLSR model.

Figure 5 shows the regression coefficients obtained from PLSR model for *F. proliferatum* concentration, namely which wavelength are highly correlated to the parameter of interest in building calibration. 

As expected, the graph shows that wavelengths at 1150, 1390–1400 nm (corresponding to fungal characteristics), and 1900 nm (related to moisture content of the matrix) have the strongest effect on the model, because they bring the most analytical information related to fungal concentration. Such absorptions confirmed results obtained by Sirisomboon, et al. [23], who isolated the same wavelengths related to fungal presence (even though in a rice matrix), or identified as fundamental by Wang, et al. [50] to discriminate fungal-damaged soybean seeds. At the moment, it is quite difficult to compare our results with those obtained in the cereal sector, where NIRS application for the healthy status of vegetal matrices has been earlier developed, because they are more interested in quantifying the presence of the mycotoxins produced by fungi than in the fungal concentration itself. It could be justified by the fact the the actual regulation in that field gives limits only for the presence of mycotoxins in kernels or flour and not for fungal concentration. Finally, at this stage of the research, NIR seems to be a promising technique to quantitatively determine fungal concentration in garlic cloves samples infected by *F. proliferatum*, because a positive correlation has been found between reference assay data and NIRS predicted results; however, at the moment we are not able to assume that NIRS could discriminate among different species of fungi developed in samples and probably altogether responsible for visual grade of infection. 

### 3.4. NIRS Model External Validation

**Figure 6:**
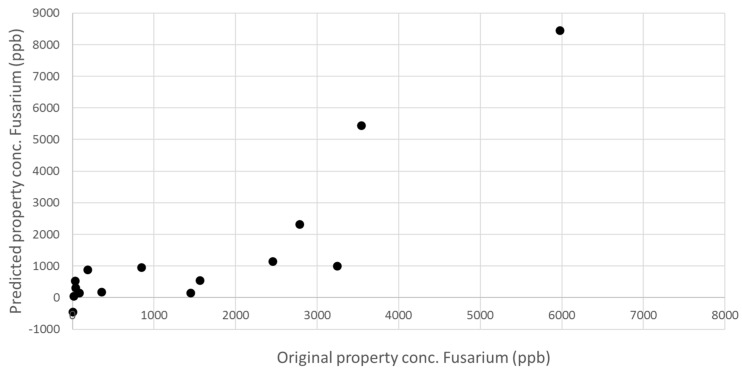
External validation of NIRS calibration model for the prediction of *F. proliferatum* concentration in 15 unknown samples of peeled garlic cloves.

**Figure 7:**
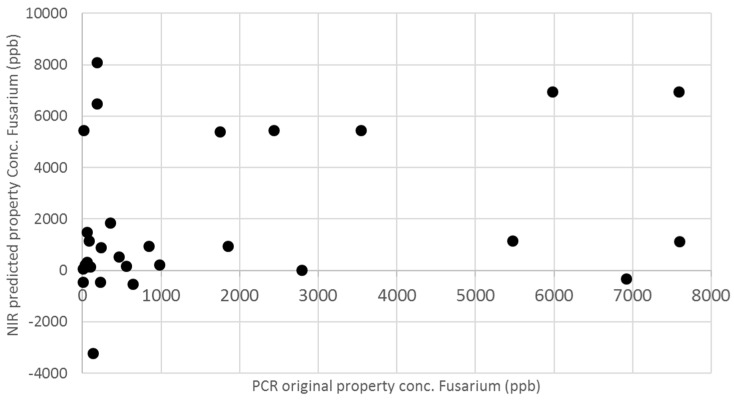
External validation of NIRS calibration model for the prediction of *F. proliferatum* concentration in 30 unknown samples of unpeeled garlic cloves.

External validation was carried out for *F. proliferatum* concentration in order to evaluate the actual predictive capability and robustness of the calibration model. An internal validation procedure cannot be considered a sufficient test, especially when NIRS signals are strongly affected by sample composition and structure, and in the case of low concentrations of parameter of interest. External validation of the model has to demonstrate the performance of the chosen model for future (unknown) samples, using an independent validation set consisting of samples that have not be used in the creation of the calibration. In particular, the calibration model was built using peeled cloves, because the main aim of the present study was to demonstrate the feasibility of NIR method proposed and principally the existence of a positive correlation between NIR spectra and fungal concentration in intact samples. Nevertheless, as expected, the possibility of predicting fungal concentration in unpeeled cloves is the future challenge of this application, because, in field, cloves used as seeds are always unpeeled. The advantage of using NIRS as an “instrumental eye” able to see through the peel (instead of people) could provide an important step forward in avoiding errors in healthy state attribution. External validation was carried out in two steps—firstly using peeled samples, so as to verify the prediction ability of the model without adding further sources of variability, and then using unpeeled samples, to get closer to the real application. Accordingly, an additional 15 peeled and 30 unpeeled garlic cloves samples were collected, as previously described, and submitted to NIRS detection and reference assays. The results of these supplementary tests have been reported in terms of NIR-predicted *F. proliferatum* concentration against the off-line analytical assay results (Figure 6 and Figure 7). 

External validation with peeled samples showed that the prediction ability of the model, even though improvable, was quite satisfactorily and demonstrated a promising predictive ability of unknown samples, with R^2^ of 0.7614 and RMSEP of 1458 ppb. Unfortunately, the external validation of unknown unpeeled samples has completely failed, and any correlation has been evidenced, indicating that the presence of peel dramatically influences the spectra signal. Assuming that validation failed due to the different sample characteristics, the subsequent step could be to integrate the 30 spectra of unpeeled samples in the calibration set and to repeat the calibration development, including the variable “presence of peel” in the model and then re-validating based on an iterative procedure to reach satisfactory prediction ability.

## 4. Conclusions

The application of NIR spectroscopy for quantitative determination of *F. proliferatum* concentration in garlic cloves at different stages of infection has been studied in this research. From the study, the NIR spectra show that a positive correlation exists and statistical parameters of calibration and external validation for peeled cloves are promising, even though further improvement of the model accuracy should be considered. NIRS is not able to predict the fungal concentration in unpeeled samples using the basic calibration developed here, and needs further study. Once properly improved, this method could be useful not only in seeding, as an automatic selection method of healthy unpeeled cloves, but also in evaluating the healthy state of garlic bulbs in post-harvest and storage. Additional samples, also characterized for other fungal species, have to be included in the model in order to investigate the capacity of NIRS to discriminate among different species.

## Figures and Tables

**Table 1:** *F. proliferatum* concentration (expressed in ppb) ranges, average values, and outlier samples grouped for grades of infection.

Grade of Infection	Range (ppb)	Average Value (ppb)	# Samples	# Outliers
1	0.34–20.68	10.51	20	6
2	18.31–79.88	49.10	20	4
3	59.76–322.13	190.94	20	2
4	345.27–1986.34	1165.80	15	2
5	2019.20–7231.15	4625.17	20	5

**Table 2:** Partial least squares regression (PLSR) results obtained for *F. proliferatum* concentration (expressed in ppb). C-set: calibration set; DW: Durbin-Watson test; RPD: ratio of performance to deviation; SEC: standard error of calibration; SECV: standard error of cross-validation; SEL: standard error of the laboratory; WL: wavelength.

Statistical Parameter	Calibration	Cross Validation
Units	ppb	ppb
SEL-reproducibility	0.10	0.10
# Samples	190	95
Outliers	0	0
Min	0.34	0.95
Max	7231.15	6541.89
SD	1391.17	1467.98
Segment (nm)	4	4
WL range/step (nm)	1000–2500/8	1000–2500/8
Pretreatments	SNV/D1	SNV/D1
Regression method	PLSR	blockwise CV
Number of factors	4	-
SEC	615.17	-
R^2^	0.829	0.774
SECV	-	717.41
NIRS repeatability	0.11	0.11
DW	2.09	1.84
C-Set Durbin–Watson in range 1.5 to 2.5?	yes	yes
Q-value	0.85	-
RPD	2.26	2.04
